# Calculation method and analysis of residual stress in the strip bending roller straightening process

**DOI:** 10.1038/s41598-024-59305-y

**Published:** 2024-04-21

**Authors:** Xiaoyu Zhu, XiaoGang Wang, Huihai Wu

**Affiliations:** Engineering Research Center Heavy Machinery Ministry of Education, Taiyuan University of Science and Technology, Taiyuan, 030024 Shanxi China

**Keywords:** Curvature integral, Bending roll straightening, Finite element, Residual stress, Blind hole method, Mechanical engineering, Mathematics and computing

## Abstract

Taking thin gauge strip as an example, the deformation process of metal strip in bending roll straightening was studied. Based on the theory of discrete, curvature integral and elastic–plastic mechanics, the strip travel trajectory of the bending roll straightening process is analyzed, and the numerical analytical calculation method of the continuous straightening process of the strip bending roll is established. The results are verified by establishing MARC finite element simulation and designing straightening experiment. The effects of yield strength, plastic rate and bending amount on residual stress after straightening were studied. In the straightening process, with the increase of the amount of bending roll, the residual strain converges to the region Γ, and with the increase of the yield strength, the region Γ decreases. With the increase of the yield strength, the amount of bending roll and the plastic rate, the wave height increases. The results of the calculation of residual stress, finite element simulation and experiment are close and the trend is consistent. The results show that the logic of the calculation method is reasonable, and the prediction error is within the scope of engineering application, which is helpful to the realization of process intelligence in the process of bending roller straightening.

## Introduction

In recent years, with the rapid development of the iron and steel industry, finished strip steel has gradually changed from a low-end product to a precision and high-end product. With the increasing demand for cold-rolled wide and thin strip steel, the quality requirements for flatness have improved^1^. The roll straightening process plays a key role in improving the quality and efficiency of cold-rolled wide and thin strip steel; this process can reduce the flatness defects and large residual stress of cold-rolled wide and thin strip steel and reduce the generation of defects and waste resources in subsequent processing and use processes^2–4^.

In the metal plate and strip production process, residual stress has been an important factor in restricting the development of plates and strips to thin and wide specification products and is also the main cause of various defects in plates and strips. The straightening theory^5^ provides theoretical support for plates with various defects, and various defective plates undergo several alternating bending elastic‒plastic deformations to flatten the plate and strip. Scholars at home and abroad have conducted much research on various aspects of residual stress formation, elimination and detection. For example, Wang Nan^6^ classified the measurement methods used at various stages, reviewed the research on residual stress measurement methods over the past five years, summarized the problems and difficulties associated with each measurement technology, and predicted future development trends. Zhang et al.^7–11^ studied the aspects of plate and strip miscellaneous rolling and heat treatment at the microscopic and macro levels and concluded that residual stress is involved in plate and strip miscellaneous rolling and heat treatment. residual stresses act as a major factor in strip wave defects, and due to the asymmetric nature of composites, residual stresses cause greater strip flatness defects. Chen et al.^12^ developed an analytical model of a roll straightening machine for strips with transverse and longitudinal wave defects. The authors separated the strips longitudinally to find the proper amount of roll bending needed to achieve equal lengths for each longitudinal section. Guan Ben^13^ predicted the internal stresses during the straightening deformation process of composite plates by establishing thermomechanical coupling based on elastic–plastic mechanics for composite plates and using discrete and differential numerical computation methods with a simulation error of 15% or less, which provided theoretical value for the production of composite plates. Zhang Min^14^ used theoretical and numerical methods and three-roll bending calculations to experimentally study the change law of residual stress and strain of the plate and strip bending and elastic recovery and found that the maximum plastic strain occurs on the surface of the plate and strip and around the contact. Doege, Jong-Bin^15–17^ et al. used the Euler–Bernoulli beam theory of a material's anisotropic and kinematic hardening modeling machine to carry out straightening process analysis. The initial assumption of the contact point between the roll and the strip was used, and then the solution was reiterated to correct for success and obtain better calculation results. Yamashita, Xue et al.^18–21^, through the use of a numerical finite element model of a strip, analyzed the strip head, tail, and curvature distribution of the straightening process and other factors but did not elucidate the nature of wave-shaped strip straightening, which is less helpful in actual production. This approach is less helpful for actual production.

Existing production operators currently rely on various yield strength and strip thickness specifications provided by straightening machine suppliers. For the production of thin-gauge high-strength plates and strips, only fine-tuning can be made through experience and trial and error, which seriously reduces efficiency and production quality. Therefore, it is necessary to analyze and solve the residual stress and curvature in the straightening process of thin and wide strip steel rolls, so as to realize the rapid setting of straightening process parameters of new specification strip steel and improve the intelligent process of the whole production and processing.

In this study, a calculation method for the curved roll leveling process is established considering the discrete resolution and curvature integral. and MSC. Along with the use of a laboratory 11-roll roll straightening machine for experimental verification, analysis of yield strength, the amount of curved rolls and other material and process parameters for the curved roll straightening process, and the curved roll straightening calculation method, the calculation speed is improved, and the calculation of the stress results and trajectory and the experimental and simulation results basically coincide with the application of the thin-gauge plate curved roll straightening process to provide a certain reference value. A certain reference value is provided for the application of the thin-gauge steel plate bending roll straightening process.

## Curvature analytical method for bending roll straightening

**Figure 1:**
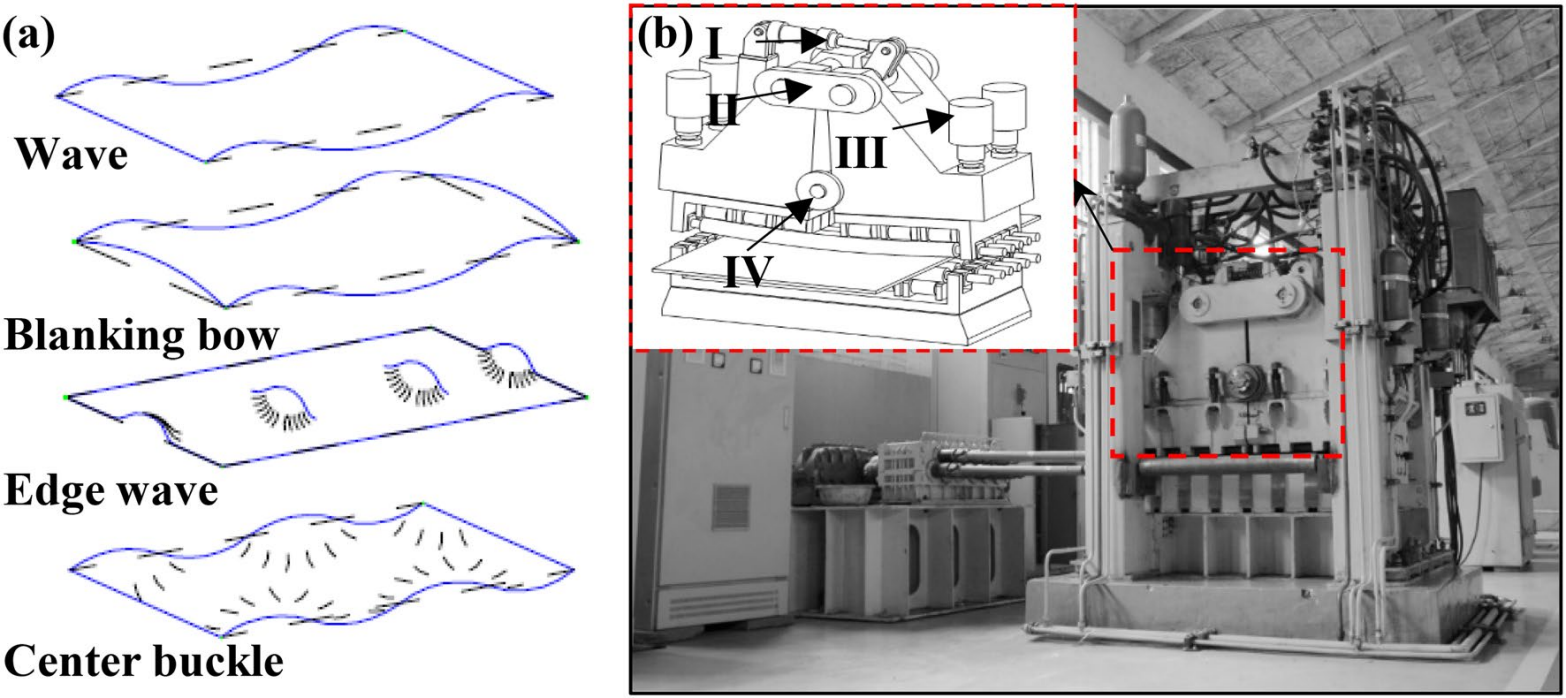
Laboratory straightening machine. (**a**) Shape defects. (**b**) Straightening machine and bending roller structure.

Bending roller straightening mainly consists of wave-shaped defective plates and strips (Fig. 1a), divided into positive bending rollers and negative bending rollers to straighten plates and strips with defects such as straightening side waves and middle waves.

In this paper, the main objective is to establish an analytical model of residual stresses in positive bending rolls by experimenting with an eleven-roll roll straightening machine and a folio bending roll structure, as shown in Fig. 1b. The overall upper roller box uses four hydraulic cylinders for overall and front-back tilt pressing down. The folio beam bends the work roll.

### Theoretical analysis under tension bending

**Figure 2:**
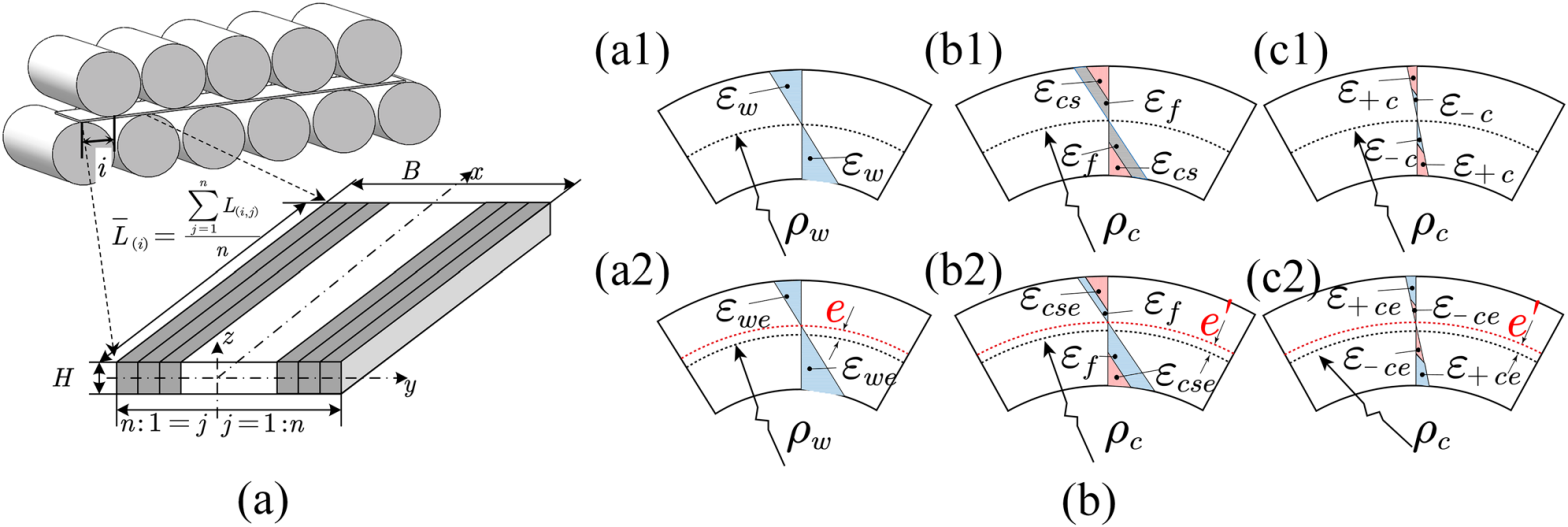
Strain and stress distributions during bending roller straightening. **a** Element division of the plate. **b** Strain and stress distribution.

To facilitate the calculation, the whole strip is divided into strip elements along the width direction, and the deformation of the straightening process is symmetrical, so it is 1 to n from the middle to both sides, as shown in Fig. 2a. In the process of bending roll straightening, due to the bending roll, the straightening reduction in each element in the width direction is different, as shown in Fig. 2a. During the whole deformation process, the length is inconsistent. According to the continuity and coordination of the material, tension is formed between the strips during the straightening process, which is different from the deformation of the entire thickness section of the flat roll straightening, as shown in Fig. 2b. Therefore, the following assumptions are made:

1. The material is an isotropic ideal elastic‒plastic material, ignoring the effects of the Bauschinger and work hardening.
2. The extrusion deformation in the thickness direction of the strip can be neglected, as can the shear stress and the normal stress in the width direction of the strip.
3. Before and after bending deformation, the normal line of the neutral surface remains unchanged

According to the whole straightening process, the average width of each element is calculated as follows:

1 $${\overline{L} }_{\left(i\right)}=\frac{\sum_{j=1}^{n}{L}_{(i,j)}}{n}$$

where ${L}_{(i,j)}$ is the length of the j-th strip element in the i-th interval, ${\overline{L} }_{(j)}$ is the average length of the bar element on the width of the plate.

According to the continuous line between the strip elements, the strain of each strip element under tension is obtained as:

2 $${\varepsilon }_{e\left(i,j\right)}=\frac{{L}_{\left(i,j\right)}-{\overline{L} }_{\left(j\right)}}{{L}_{\left(i,j\right)}}$$

where ${\varepsilon }_{e\left(i,j\right)}$ is tensile strain.

As shown in the Fig. 2a1. Assuming that the curvature of the j-th element in the straightening process is $k{\left(x\right)}_{\left(i,j\right)}$, the radius of curvature is R, and the deformation at the height $z$ from the neutral layer can be obtained.

3 $${\varepsilon }_{w\left(i,j\right)}=\left(R+z\right)k{\left(x\right)}_{\left(i,j\right)}-Rk{\left(x\right)}_{\left(i,j\right)}=zk{\left(x\right)}_{\left(i,j\right)}$$

where ${\varepsilon }_{w\left(i,j\right)}$ is bending strain, $z$ is Distance from the neutral layer, $k{\left(x\right)}_{\left(i,j\right)}$ is the curvature function of the j-element in the interval from the $i$-roll to the $i+1$-roll.

According to Formulas (1) ~ (3), the following can be obtained for the case of tensile strain:

4 $${\varepsilon }_{we\left(i,j\right)}=zk{\left(x\right)}_{\left(i,j\right)}+{\varepsilon }_{e\left(i,j\right)}$$

where ${\varepsilon }_{we\left(i,j\right)}$ is Total strain under tension and bending moment,

The strain of the plate fiber is lower than the yield point according to the following equation: (4) derived from Hooke's law:

5 $$\sigma_{{z\left( {i,j} \right)}} \left( x \right) = \left\{ {\begin{array}{*{20}c} {E\left( {zk\left( x \right)_{{\left( {i,j} \right)}} + \varepsilon_{{e\left( {i,j} \right)}} } \right)\left( {\varepsilon_{{we\left( {i,j} \right)}} > \varepsilon_{s} } \right)} \\ {\sigma_{s} \left( {\varepsilon_{{we\left( {i,j} \right)}} \ge \varepsilon_{s} } \right)} \\ \end{array} } \right.$$

where ${\sigma }_{s}$ is yield stress, ${\varepsilon }_{s}$ is yield strain.

According to the stress distribution on the section of the strip ${\sigma }_{z\left(i,j\right)}\left(x\right)$, the j-element can be solved, and the straightening area bending moment ${M}_{\left(i,j\right)}\left(x\right)$ is:

6 $${M}_{\left(i,j\right)}\left(x\right)={\int }_\frac{Bj}{2n}^{\frac{B\left(j+1\right)}{2n}}{\int }_{-\frac{H}{2}}^\frac{H}{2}{\sigma }_{z\left(i,j\right)}\left(x\right)zdzdy$$

According to elastic–plastic mechanics, the strain changes during the unloading process of the strip are shown in Fig. 2a1–b2; according to the continuity of the material, under the conditions of no tension and tension, The state after stress balance is shown in Fig. 2c1,c2, and satisfies the equilibrium condition:

7 $$\left\{\begin{array}{c}{\int }_\frac{Bj}{2n}^{\frac{B\left(j+1\right)}{2n}}{\int }_{-\frac{H}{2}}^\frac{H}{2}\sigma {\left(x\right)}_{ce\left(i,j\right)}zdzdy=0\\ {\int }_{-\frac{H}{2}}^\frac{H}{2}\sigma {\left(x\right)}_{ce\left(i,j\right)}zdz=0\end{array}\right.$$

### Bending roll calculation model based on the curvature integration method

**Figure 3:**
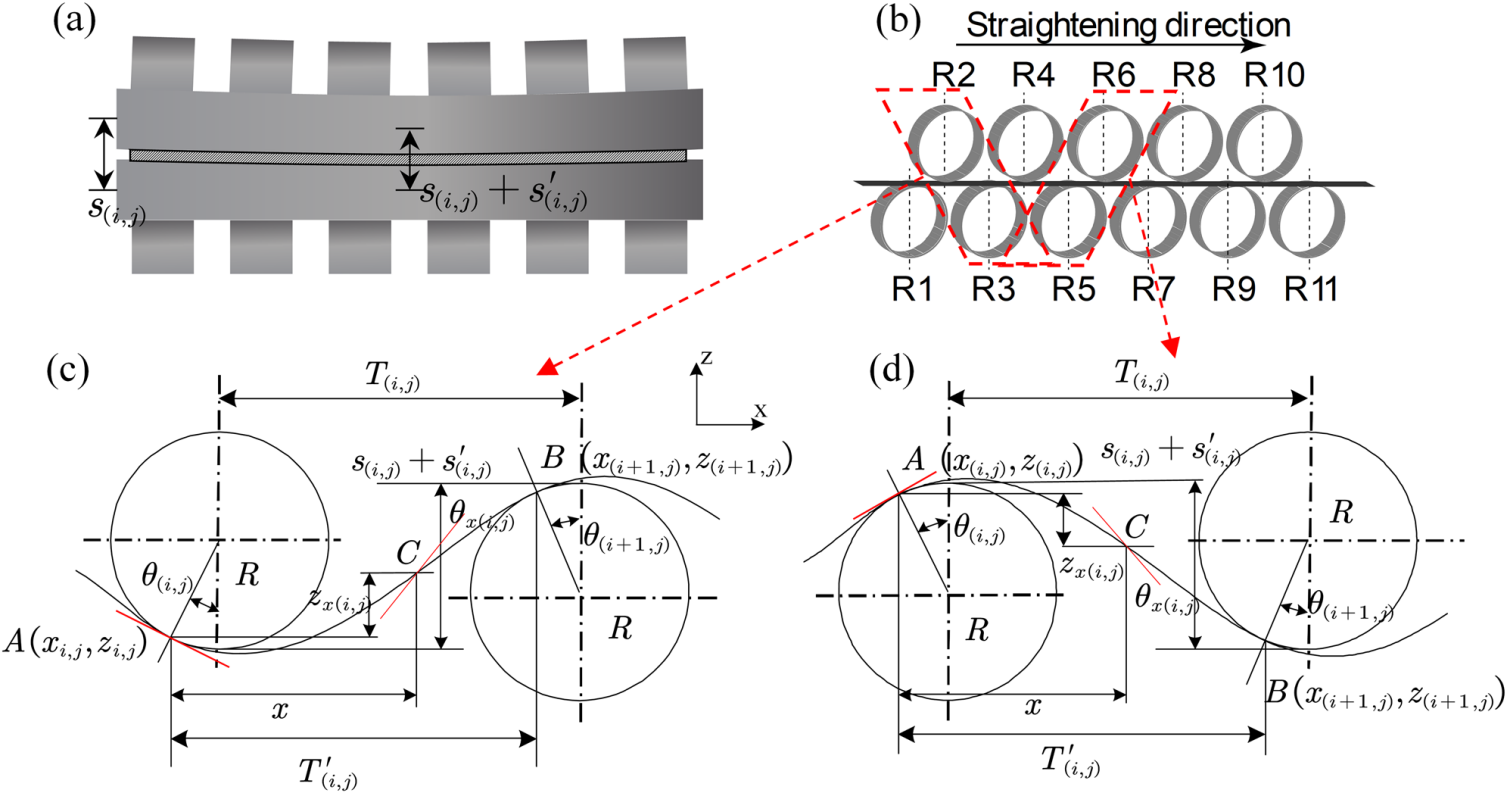
Analysis diagram of the curvature for bending roller straightening. (**a**) Bending roll reduction diagram. (**b**) Strip straightening process. (**c**) The first roll is the upper roll. (**d**) The first roll is the lower roll.

During the straightening process of the bending roller, under the action of the bending roller, different reduction amounts ${s}_{\left(i,j\right)}$ is displayed along the plate width direction, and the working rollers of the straightening machine are staggered, as shown in Fig. 3a,b. The following contact points and geometric parameters for each straightening interval are specified as follows:

1. The contact points of the $i$ and $i+1$ straightening rollers are $A\left({x}_{i,j},{z}_{i,j}\right)$ and $B\left({x}_{\left(i+1,j\right)},{z}_{\left(i+1,j\right)}\right)$, respectively, and the distance between them is ${T}_{\left(i,j\right)}^{\mathrm{^{\prime}}}$
2. The angle between the tangent line at any point C between A and B and the positive direction of the x-axis is less than 90°, which is positive, and greater than 90°, which is negative.
3. According to the section $i$-straightening area provisions, the upper roll downward is '−', and the lower roll upward is ' + '.

Taking the first roll as the upper roll in Fig. 3c as an example, according to geometric relations, the distances ${T}_{\left(i,j\right)}^{\mathrm{^{\prime}}}$ and ${\delta }_{\left(i.j\right)}$ between the contact points of the $i$-straightening area and the j-element are:

8 $$\left\{\begin{array}{c}{T}_{\left(i,j\right)}^{\mathrm{^{\prime}}}=\frac{T}{2}-{R}_{i}sin{\theta }_{\left(i,j\right)}-{R}_{i+1}sin{\theta }_{\left(i+1,j\right)}\\ {\delta }_{\left(i,j\right)}={s}_{\left(i,j\right)}+{s}_{\left(i,j\right)}^{\mathrm{^{\prime}}}-R\left(\left(1-{\text{cos}}\left({\theta }_{\left(i,j\right)}\right)\right)+\left(1-{\text{cos}}\left({\theta }_{\left(i+1,j\right)}\right)\right)\right)\end{array}\right.$$

where $T$ is the design roller distance for the straightening machine. $R$ is the radius of the rollers, ${\theta }_{\left(i,j\right)}$ and ${\theta }_{\left(i+1,j\right)}$ are defined as the contact angles at the contact points, ${s}_{\left(i,j\right)}$ is the amount of reduction in the $i$-straightening area, and ${s}_{\left(i.j\right)}^{\mathrm{^{\prime}}}$ is the amount of reduction in the j-element.

Assuming that for the j-th element, the curvature in the $i$-straightening area is $k{\left(x\right)}_{\left(i,j\right)}$; according to the curvature integral, the first and second integrals are the angular change and the coordinate change, respectively, and can be obtained:

9 $$\left\{\begin{array}{c}\theta {\left(x\right)}_{\left(i,j\right)}={\int }_{0}^{x}k{\left(x\right)}_{\left(i,j\right)}dx+{C}_{1\left(i,j\right)}\\ z{\left(x\right)}_{\left(i,j\right)}={\int }_{0}^{x}{\int }_{0}^{x}k{\left(x\right)}_{\left(i,j\right)}dxdx+{C}_{1\left(i,j\right)}x+{C}_{2\left(i,j\right)}\end{array}\right.$$

where ${C}_{1\left(i,j\right)}$ and ${C}_{2\left(i,j\right)}$ are unknown variables, $\theta {\left(x\right)}_{\left(i,j\right)}$ and $z{\left(x\right)}_{\left(i,j\right)}$ are the variations in the contact angle and coordinate.

The boundary conditions of formula (9) are as follows:

10 $$\left\{\begin{array}{c}\begin{array}{c}{\left.\theta {\left(x\right)}_{\left(i,j\right)}\right|}_{x=0}={\theta }_{\left(i,j\right)}\\ {\left.z{\left(x\right)}_{\left(i,j\right)}\right|}_{x=0}=0\end{array}\left(x=0\right)\\ \begin{array}{c}{\left.\theta {\left(x\right)}_{\left(i,j\right)}\right|}_{{T}_{\left(i,j\right)}^{\mathrm{^{\prime}}}}={\theta }_{\left(i+1,j\right)}\\ {\left.z{\left(x\right)}_{\left(i,j\right)}\right|}_{x={T}_{\left(i,j\right)}^{\mathrm{^{\prime}}}}={\delta }_{\left(i.j\right)}\end{array}\left(x={T}_{\left(i,j\right)}^{\mathrm{^{\prime}}}\right)\end{array}\right.$$

Therefore, ${C}_{1\left(i,j\right)}$ and ${C}_{2\left(i,j\right)}$ can be obtained by taking into account formula (9).

11 $$\left\{\begin{array}{c}{C}_{1\left(i,j\right)}=\theta {\left(x\right)}_{\left(i,j\right)}\\ {C}_{2\left(i,j\right)}=0\end{array}\right.$$

Similarly, when the first roll is the lower roll (as shown in Fig. 3d), ${T}_{\left(i,j\right)}^{\mathrm{^{\prime}}}, {\delta }_{\left(i.j\right)}, {C}_{1\left(i,j\right)}, {C}_{2\left(i,j\right)}$ are obtained:

12 $$\left\{\begin{array}{c}{T}_{\left(i,j\right)}^{\mathrm{^{\prime}}}=\frac{T}{2}+{R}_{i}sin{\theta }_{\left(i,j\right)}+{R}_{i+1}sin{\theta }_{\left(i+1,j\right)}\\ {\delta }_{\left(i,j\right)}={s}_{\left(i,j\right)}+{s}_{\left(i,j\right)}^{\mathrm{^{\prime}}}-R\left(\left(1-{\text{cos}}\left(\theta {\left(x\right)}_{\left(i,j\right)}\right)\right)+\left(1-{\text{cos}}\left(\theta {\left(x\right)}_{\left(i+1,j\right)}\right)\right)\right)\\ {C}_{1\left(i,j\right)}=\theta {\left(x\right)}_{\left(i,j\right)}\\ {C}_{2\left(i,j\right)}=0\end{array}\right.$$

Thus, according to Formulas (8) ~ (12), the core equation of the curvature integral model of the whole bending roll straightening process is

13 $$\left\{\begin{array}{c}{\theta }_{\left(i+1,j\right)}={\int }_{0}^{{T}_{\left(i,j\right)}^{\mathrm{^{\prime}}}}k{\left(x\right)}_{\left(i,j\right)}dx+{\theta }_{\left(i,j\right)}\\ {\delta }_{\left(i.j\right)}={\int }_{0}^{{T}_{\left(i,j\right)}^{\mathrm{^{\prime}}}}{\int }_{0}^{{T}_{\left(i,j\right)}^{\mathrm{^{\prime}}}}k{\left(x\right)}_{\left(i,j\right)}dxdx\mp {\theta }_{\left(i,j\right)}x+R\left(\left(1-{\text{cos}}{\theta }_{\left(i,j\right)}\right)+\left(1-{\text{cos}}{\theta }_{\left(i+1,j\right)}\right)\right)-{s}_{\left(i.j\right)}^{\mathrm{^{\prime}}}\end{array}\right.$$

where '−' is the first roll for the upper roll and the ' + ' first roll is the lower roll.

In summary, the nonlinear equations of the straightening process of each element can be established by the nonlinear Eq. (13).

**Figure 4:**
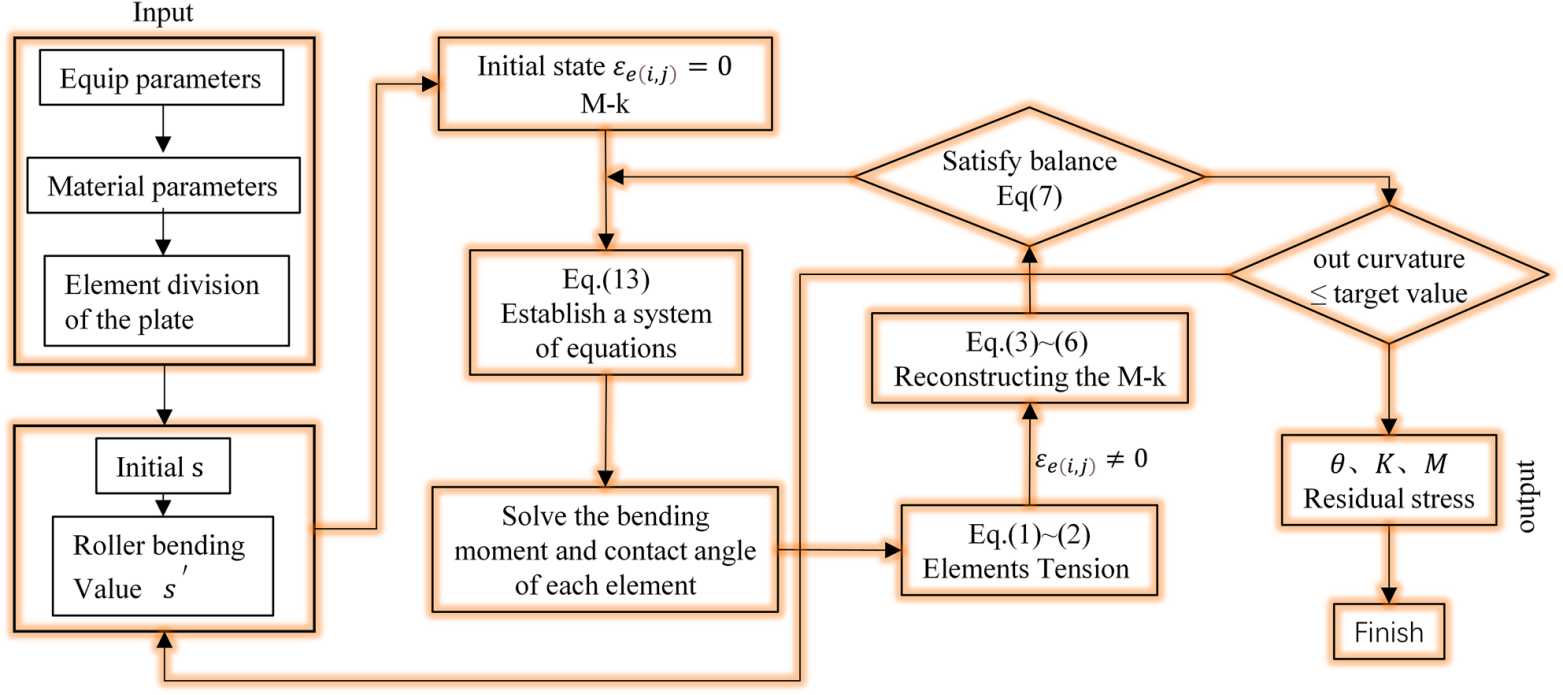
Procedure of building explicit curvature integration equations.

Figure 4 summarizes the process of calculation model of bending roll straightening process. First. The parameters such as the structure of the straightening machine, the physical properties of the material and the number of strip elements are input. The second step is to input the first roll reduction and bending amount of the straightening machine. The third step is to construct the curvature integral equation set Eq. (13) of the whole strip element straightening process with the non-tension stress superposition bending moment–curvature as the initial solution condition. Then the least square method is used to solve the equation, and then the tension is calculated according to Eqs. (1)–(2). Equations (3)–(6) re-solve the relationship between bending moment and curvature. According to Eq. (7) as the equilibrium condition, and through the flatness judgment as feedback, the straightening accuracy is improved. Finally, the contact angle, curvature, bending moment and residual stress are output to meet the industrial requirements. The optimization iterative algorithm is implemented in MATLAB.

## Numerical simulation and calculation results

### Finite element model

**Figure 5:**
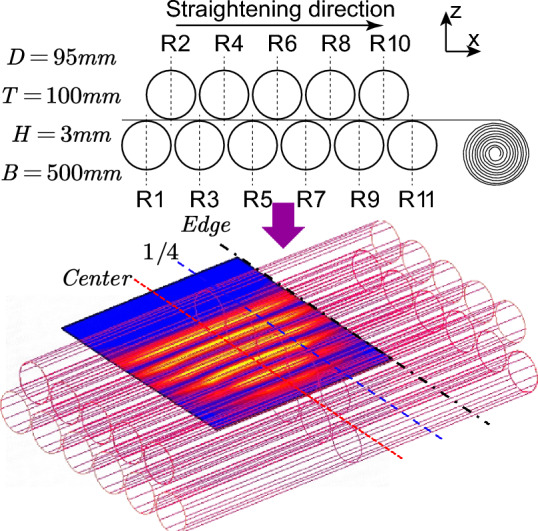
Simulation model of the bending roll straightening process.

In order to verify the reliability of the analytical model and process of transverse residual stress, the finite element software MSC. Marc is used to establish the three-dimensional model shown in Fig. 5. The 11 straightening rollers are set as rigid bodies, and the strip is set as a deformable body. The 8-node reduced integral unit is used. The material is a rational elastic–plastic material. The plate width unit size is 10 mm, and the length unit size is 5 mm. The thickness unit size is 0.5 mm, a total of 30,000 units, and the friction coefficient is set to 0.15. Considering the symmetry of the strip deformation, in order to consider the calculation cost, the symmetry constraint is added in the y direction of the middle node of the plate width, and the x and z directions are constrained by the contact relationship between the strip and the roll. The analysis process uses quasi-static analysis, large deformation, and updated Lagrange method.

To study the influence of the plate yield strength and bending roll amount on the straightening process of a plate bending roll, this paper uses the control variable method to carry out cross-simulation calculations and experiments on different yield strengths and bending roll amounts. The position curves of the Center, 1/4 and Edge in the width direction are extracted. The simulation results are compared and analyzed with the straightening calculation trajectory, and the strain in the width direction after straightening is compared and analyzed to verify the accuracy of the calculation.

### Straightening path analysis

**Figure 6:**
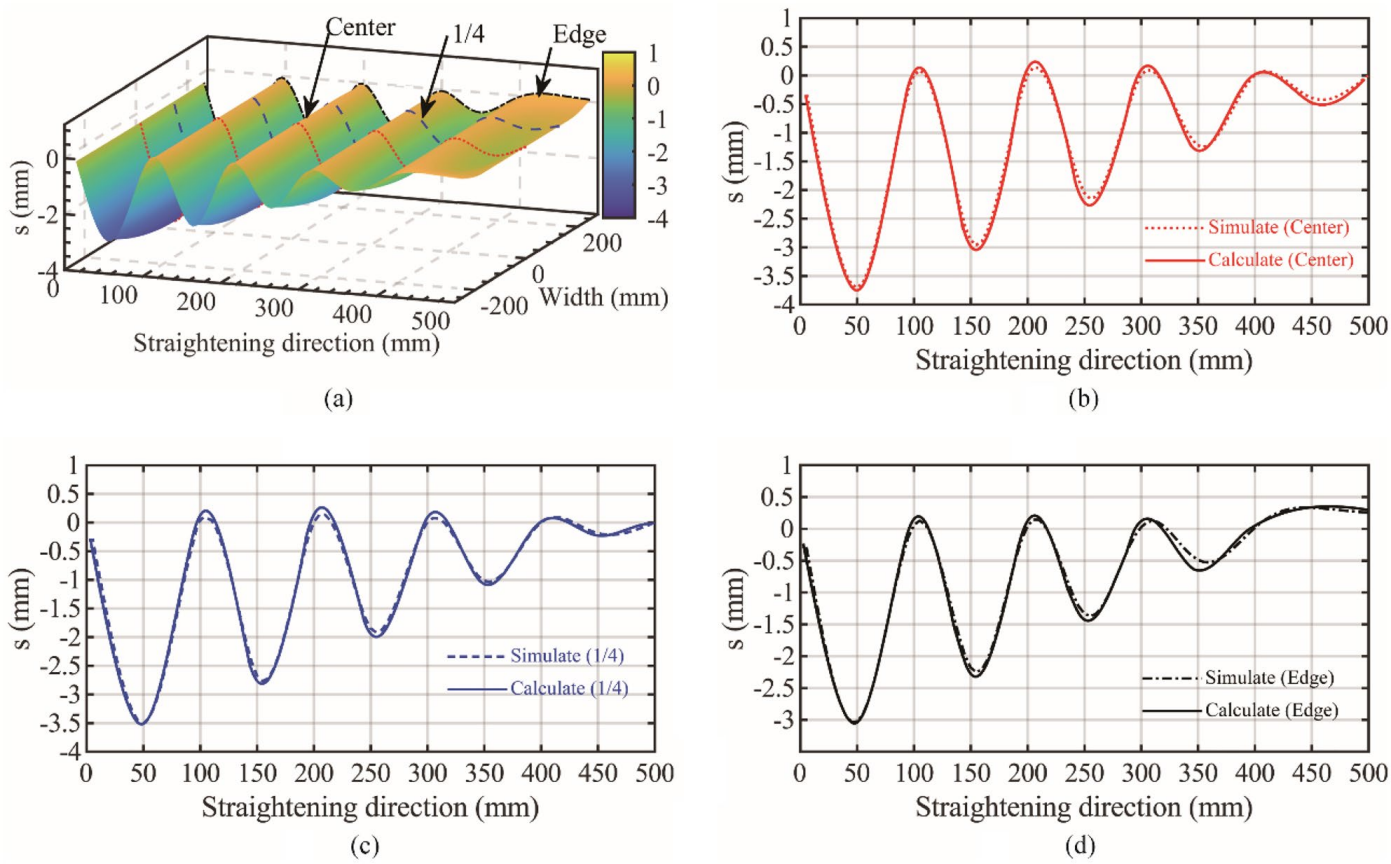
Straightening process simulation and calculation trajectory comparison. (**a**) Simulation result extraction. (**b**) Center contrast trajectory. (**c**) 1/4 contrast trajectory. (**d**) Edge contrast trajectory.

Taking 1000 MPa, a bending roll of 3 mm and a plasticity rate of 66.7% as an example, the surface results of the bending roll straightening process were extracted, as shown in Fig. 6a. The three simulated trajectory curves of the Center, 1/4 and Edge trajectories are compared with the corresponding theoretical trajectories. The results are shown in Fig. 6b–d. The trajectory trend is almost consistent, and the error is small. Considering the calculation time cost, meshing and other reasons, the error is within a reasonable range.

### Analysis of the strain results

**Figure 7:**
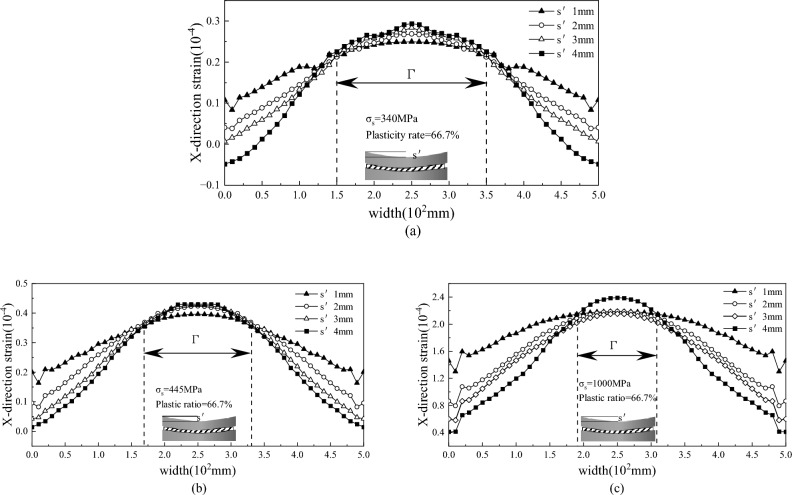
Residual strain under different yield and bending amounts after straightening for (**a**) 340 MPa, (**b**) 445 MPa, and (**c**) 1000 MPa.

**Figure 8:**
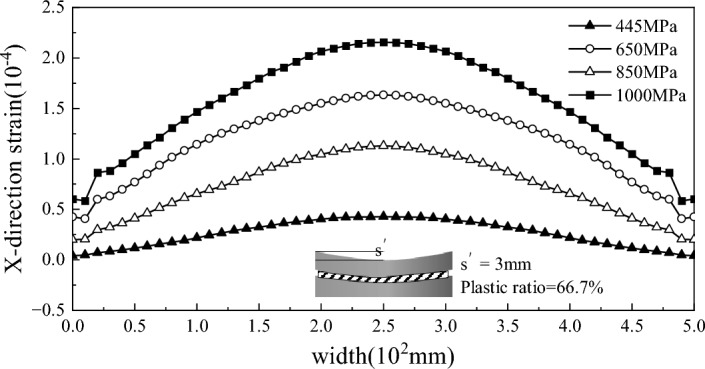
Residual strain of strips with different yield strengths after straightening.

**Figure 9:**
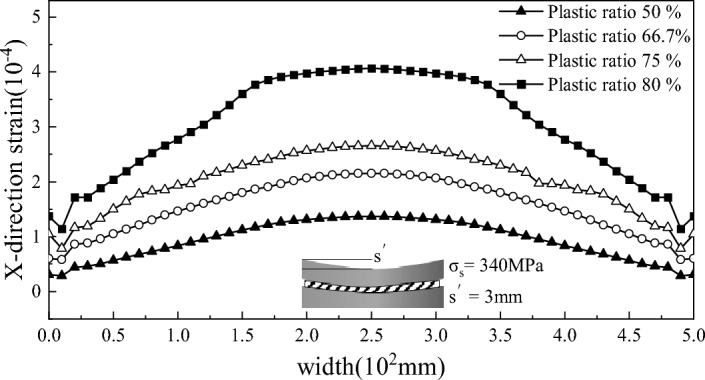
Residual strain of the strip after straightening with different plastic rates.

In order to analyze the different yield strength and plastic rate, as well as the deformation of the bending roll straightening process, MSC. Marc was used to establish a variety of finite element models, and the data of the finite element simulation results were extracted and plotted, as shown in Figs. 7, 8 and 9.

As shown in Fig. 7a–c, under the same plastic rate of bending straightening process, the strain distribution of different bending amounts along the width direction obeys the quadratic distribution, and the residual strain convergence interval of the quadratic distribution of the same strength strip under different bending amounts is $\Gamma$, and $\Gamma$ decreases with the increase of yield strength. This case also verifies the problem that the wave-shaped plate is difficult to straighten in the case of high-strength plate.

To study the deformation of sheets with different yield strengths during the roll bending and straightening process under the same plasticity rate of 66.7% and the same roll bending amount of 3 mm, finite element models of 455 MPa, 650 MPa, 850 MPa and 1000 MPa were established. The strain is shown in Fig. 8. As the yield strength increases, the residual strain after bending roll straightening increases, which also reflects why high-strength plates require a large amount of roll bending to eliminate corrugated strip defects.

As shown in Fig. 9, to study the influence of the plasticity rate, the 340 MPa plate was simulated using a 3 mm roll bending amount, and the plasticity rates were 50%, 66.7%, 75% and 80%. The figure shows that the plasticity rate has an impact on the consistency with the influence of residual strain; as the plasticity rate increases, the wavy strain generated after straightening increases. However, at an 80% plasticity rate, the strain exhibited a wavy shape, and the plate belt tended to buckle. Verification According to the actual engineering process, although increasing the plasticity rate can achieve straightening of the wave shape, the amount of roll bending or reduction is too large, resulting in the generation of new wave shapes.

## Straightening experiment

### Residual stress test

**Figure 10:**
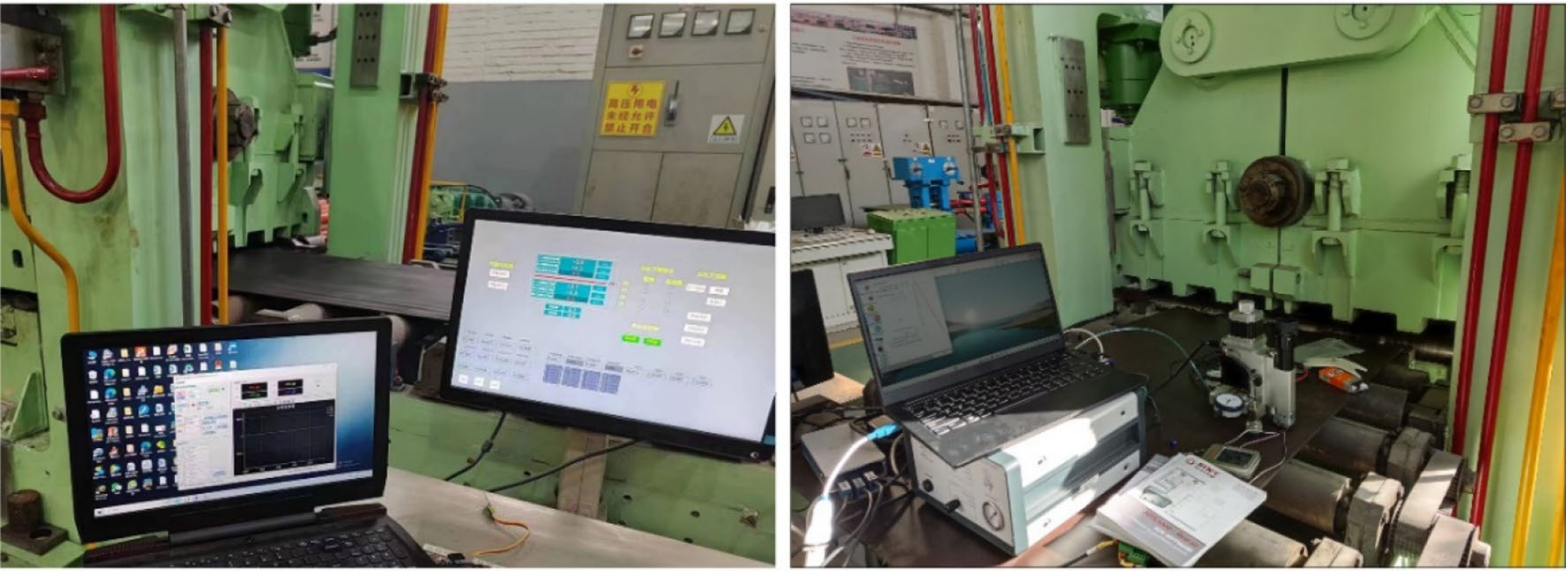
Straightening and residual stress acquisition experiment.

In order to verify the accuracy of the calculation method and the finite element results. Bending roll straightening and residual stress collection experiments were performed on a 3 mm yield strength of 340 MPa, as shown in Fig. 10. In order to be close to the current situation of iron and steel production, the production process is set according to the size of the straightening machine in the laboratory. The bending rolls are 1 mm and 2 mm respectively, and the plasticity rate is 75%. The residual stress is detected by the blind hole method^6^. The equipment is the Italian blind hole method automatic residual stress detector. The model is RESTAN-MTS3000. The plate width is divided into 14 equal parts, and 13 points are selected for data measurement. In order to reduce the error, each point is measured 5 times to get the average value.

### Comparison of calculations, experiments and simulations

**Figure 11:**
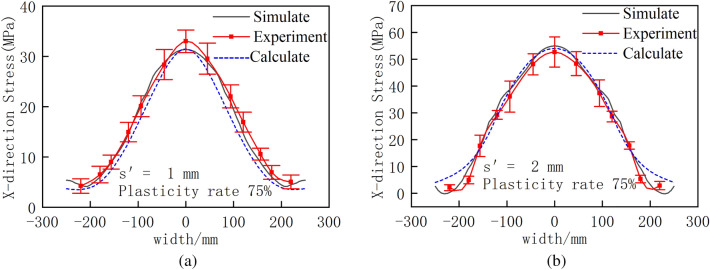
Comparison of calculations, experiments and simulations. (**a**) roller bending value of 1 mm (**b**) roller bending value of 2 mm.

As shown in Fig. 11, the experimental, calculation and simulation results all obey the quadratic distribution form. The results of the calculation method and the finite element simulation are consistent with the experimental results. Due to the biting process of the straightening experiment, the plate is not centered, which causes an offset, and stress asymmetry occurs; however, the stress is within the error range, meeting the needs of industrial applications.

In summary, the roll bending amount and plasticity rate play important roles in straightening the roll bending of corrugated defective strips. Both the experimental and simulation results verify the accuracy of the numerical calculations. Due to the high efficiency of the numerical calculation, this approach is better than simulation because it greatly reduces the calculation time, allows quick analysis of the distribution of residual stress after straightening, and plays a good role in predicting the plate shape.

## Conclusion

In this paper, a new method for analyzing the residual stress in the straightening process of a bending roller is proposed. Based on the above results, the following conclusions can be drawn:

1. A simulation model of bending roll straightening is established, and the deformation of plates with different yield strengths is analyzed by the bending roll amount and plasticity rate. Under the same plasticity rate, without a yield strength plate, with increasing bending roll, the wave strain converges to the interval $\Gamma$, and with increasing yield strength, $\Gamma$ decreases. For the same yield strength plate, with increasing roll bending amount and plasticity rate, the strain wave shape increases.
2. Through discrete analysis and curvature integration, a numerical calculation method for the bending roller straightening process was established, and the proposed analytical solution results were compared with the simulation results and experimental results. The straightening process trajectories of the theoretical calculation and simulation results were similar, and the calculation and residual stress errors between the experiment and simulation were small, which verifies the correctness of the calculation logic.
3. The analytical method of residual stress in the straightening process of bending roll curvature integral proposed by has a short solution time, which can quickly predict the straightening process of bending roll, reduce the error of operators, reduce the waste of resources, and help to realize the intelligent process parameters of the straightening process of wavy plate.

## Data Availability

The project is currently ongoing and the calculation procedures cannot be published. All data generated or analyzed during this research are included in the images of this published article. Detailed data from the figures generated and/or analyzed during the current study are available from the corresponding author on reasonable request.
